# Gut Microbiome Was Highly Related to the Regulation of Metabolism in Lung Adenocarcinoma Patients

**DOI:** 10.3389/fonc.2022.790467

**Published:** 2022-05-03

**Authors:** Sheng Wang, Huachun Chen, Huizhen Yang, Kejin Zhou, Fan Bai, Xiaoyu Wu, Hanwen Xu

**Affiliations:** Department of Respiratory, Jinhua Guangfu Hospital, Jinhua, China

**Keywords:** gut microbiome, metabolism, lung adenocarcinoma, gut-lung axis, diagnosis

## Abstract

**Background:**

Lung adenocarcinoma (LUAD) is one of the most predominant subtypes of lung cancer. The gut microbiome plays a vital role in the pathophysiological processes of various diseases, including cancers.

**Methods:**

In the study, 100 individuals were enrolled. In total 75 stool and blood samples were analyzed with 16s-rRNA gene sequencing and metabolomics (30 from healthy individuals (H); 45 from LUAD patients). In addition, 25 stool samples were analyzed with metagenomics (10 from H; 15 from LUAD). The linear discriminant analysis (LDA) effect size (LefSe) and logistic regression analysis were applied to identify biomarkers’ taxa and develop a diagnostic model. The diagnostic power of the model was estimated with the receiver operating characteristic curve (ROC) by comparing the area under the ROC (AUC). The correlation between biomarker’s taxa and metabolites was calculated using the Spearman analysis.

**Results:**

The α and β diversity demonstrated the composition and structure of the gut microbiome in LUAD patients were different from those in healthy people. The top three abundance of genera were Bacteroides (25.06%), Faecalibacterium (11.00%), and Prevotella (5.94%). The LefSe and logistic regression analysis identified three biomarker taxa (Bacteroides, Pseudomonas, and Ruminococcus gnavus group) and constructed a diagnostic model. The AUCs of the diagnostic model in 16s-rRNA gene sequencing and metagenomics were 0.852 and 0.841, respectively. A total of 102 plasma metabolites were highly related to those three biomarkers’ taxa. Seven metabolic pathways were enriched by 102 plasma metabolites, including the Pentose phosphate pathway, Glutathione metabolism.

**Conclusions:**

In LUAD patients, the gut microbiome profile has significantly changed. We used three biomarkers taxa to develop a diagnostic model, which was accurate and suitable for the diagnosis of LUAD. Gut microbes, especially those three biomarkers’ taxa, may participate in regulating metabolism-related pathways in LUAD patients, such as the pentose phosphate pathway and glutathione metabolism.

## Introduction

Lung cancer (LC) is one of the most commonly diagnosed malignancies and the leading origin of disease-related mortality throughout the world (1). It was estimated that, in 2020, more than 2.2 million new cases were diagnosed as LC, and over 1.8 million people died of LC globally (2, 3). According to histopathological differences, LC could be divided into Non-small cell lung cancer (NSCLC) and Small cell lung cancer (SCLC). Lung adenocarcinoma (LUAD) is one of the most predominant subtypes of NSCLC, accounting for approximately 40% of cases of LC (3). Surgery, chemotherapy, targeted therapy, and immunotherapy are the main treatments for LC and are developing continuously, which have greatly improved the long-term survival rate of LC patients (4, 5). However, the 5-year survival rate of LC patients is still less than 20% (6, 7). To make matters worse, nearly 75% of LC patients are in the advanced stage at the initial visit, and for these the 5-year survival rate is only 2.8%-14.6% (6, 7). Therefore, it is urgent to identify the underlying mechanisms, diagnostic biomarkers, and therapeutic targets of LC, which is helpful for the diagnosis and therapy of LC.

The intestinal tract is the main place for digestion and absorption. It is also an important endocrine and immune organ, playing a crucial role in maintaining the normal physiological function of the body (8). In the past decade, one inspiring finding in medicine was the vital role that the gut microbiome played in the pathophysiological processes of various diseases, such as neurodegenerative diseases, metabolic diseases, immune and inflammatory diseases, mental diseases, and cancer (9–11). The gut microbiome refers to the microbial community living in the intestine, including bacteria, fungi, protozoa, and viruses, counting over ten times the number of total host cells (12). In the long-term evolution process, the gut microbiome and the host have formed a mutually beneficial symbiotic relationship: on the one hand, the gut microbiome obtains nutrients necessary for survival from the host; on the other hand, it could exert a variety of biological functions to contribute to the host digest and absorb nutrients, activate and stabilize the immune system (12, 13).

In recent years, a novel viewpoint, “Gut-lung axis”, was proposed, which means the long-distance cross-talk between lung and intestine (14). Numerous studies have shown that the gut microbiome could influence lung homeostasis and susceptibility to lung diseases by regulating the metabolic, endocrine, and immune system (15). Although few kinds of research have been reported on the characteristics of the gut microbiome in LC patients, similar acknowledgments have been achieved. Compared with healthy individuals, the evenness and richness of the gut microbiome in LC patients have changed significantly, with a decrease in the relative abundance of beneficial bacteria and an increase in harmful bacteria (16). In addition, studies have demonstrated that the changes in the composition and structure of the gut microbiome also evidently affect the therapeutic effects of LC patients (17, 18).

In the study, we collected 60 stool samples from LUAD patients. Then, the stool samples were subjected to 16s-rRNA gene sequencing or metagenomics to investigate the gut microbiome profile in LUAD and construct a diagnostic model for LUAD. Next, we predicted the functions of the gut microbiome through bioinformatics and found the gut microbiome enriched several metabolic-related pathways. Finally, we verified the results by metabonomics.

## Materials and Methods

### Patients

The study was permitted by the ethics committee of Guangfu hospital. In the study, 100 participants were collected, including 40 healthy individuals (H) and 60 LUAD patients. All participants were from Guangfu hospital and signed relevantly informed consent. Among them, 30 healthy individuals and 45 LUAD patients were analyzed with 16s-rRNA gene sequencing and metabonomics; the others with metagenomics. The basic information of all subjects was presented in **Table 1**.

**Table 1:** The basic information of all participants.

	16s-rRNA gene/Metabonomics			Metagenomics		
	H(n=30)	LUAD (n=45)	P	H(n=10)	LUAD (n=15)	P
Age	58.5 ± 7.02	59.7 ± 10.20	0.453	57.9 ± 8.02	60.1 ± 11.10	0.632
Gender (F/M)	14/16	21/24	0.123	5/5	8/7	0.342
Height (cm)	166.1 ± 5.52	164.5 ± 6.74	0.263	164.7 ± 6.42	163.5 ± 7.74	0.462
Weight (kg)	62.2 ± 11.13	60.2 ± 10.81	0.376	61.8 ± 11.43	58.9 ± 11.81	0.453
BMI	21.1 ± 2.63	20.10 ± 3.01	0.563	20.9 ± 3.46	21.34 ± 4.01	0.745
Site (Right/Left)	–	23/22	–	–	6/9	–
TNM (I,II/III,IV)	–	20/25	–	–	7/8	–
Size (cm)	–	2.5 ± 1.91	–	–	3.1 ± 2.34	–
Lymph node (N0/N1-3)	–	14/31	–	–	6/9	–
Metastasis (M0/M1)	–	30/15	–	–	11/4	–
CEA (ng/ml)	–	6.1 ± 9.72	–	–	7.3 ± 7.62	–
NSE (ng/ml)	–	19.1 ± 30.53	–	–	9.9 ± 18.46	–
CYFRA211 (ng/ml)	–	4.6 ± 6.74	–	–	8.4 ± 9.35	–
CA153 (U/ml)	–	5.9 ± 2.42	–	–	4.3 ± 3.13	–
CA125 (U/ml)	–	3.2 ± 3.69	–	–	4.1 ± 2.15	–

H, healthy individual; LUAD, Lung adenocarcinoma; F, Female; M, Male; CEA, Carcinoembryonic antigen; NSE, Neuron-specific enolase. CYFRA211, Tokeratin 19 fragment; CA125; Carbohydrate antigen 125; CA153, Carbohydrate antigen 153.

Patients were diagnosed as LUAD according to the TNM staging system (8th edition) and judged by two pathologists. The inclusion and exclusion criteria were as follows:

Inclusion criteria: 1) 18 ≤ Age ≤ 75 years; 2) Pathological diagnosis or cytological diagnosis; 3) The patients have never been treated, including surgery, chemotherapy, radiotherapy, targeted therapy, and immunotherapy; 4) KPS ≥ 60 and ECOG ≤ 2; 5) Life expectancy ≥ 6 months.

Exclusion criteria: 1) With other acute and chronic diseases influencing the composition of the gut microbiome, such as metabolic diseases, mental illness, other cancers; 2) Women were in pregnancy or breastfeeding; 3) Abnormal blood test, including white blood cell < 4*109/L, neutrophils < 2*109/L, hemoglobin < 100 g/L, platelet < 100 g/L, Hemobilirubin > 1.5 ULN (upper limit of normal value), ALT, AST > 2.5 ULN, and serum creatinine >1.5 ULN; 4) Treated with antibiotics in the past 3 months.

### Stool and Blood Sample Collection

Fecal samples were collected using Fecal Collection Kit (Beyotime, China). DNA was extracted from fecal samples with E.Z.N.A. ^®^Stool DNA Kit (Omega, USA) and eluted with 50 μL of elution buffer, and stored at −80°C.

Blood samples of 5 mL were gathered from a subject with an anticoagulation tube (Ethylene Diamine Tetraacetic Acid) and then centrifuged at 3500 rpm (15 min, 4°C). The supernatant was collected as the plasma samples and stored at − 80°C.

### 16s-rRNA Gene Sequencing

Seventy-five stool samples (30 H; 45 LUAD) were analyzed with 16s-rRNA gene sequencing. The primers that targeted the V3-V4 region of 16s-rRNA gene were as follows:

341F: 5′-CCTACGGGNGGCWG- CAG-3′;
805R: 5′-GACTACHVGGGTATCTAATCC-3′.

As described previously (19), PCR amplification was performed. The PCR products were then purified and quantified with AMPure XT beads (Beckman Coulter Genomics, USA) and Qubit (Invitrogen, USA), respectively. The size and quantity of the PCR products were evaluated using the Library Quantification Kit (Kapa Biosciences, USA), and to develop the amplicon pools following the manufacturer’s recommendations, which were applied for sequencing with the Illumina NovaSeq platform (Illumina, USA). Then, 250 bp paired-end reads were generated and merged to raw reads with FLASH. The high-quality clean tags were produced through screening raw reads using the fqtrim (v0.94). Then, we removed repeated sequences and chimeric sequences with Vsearch software (v2.3.4) and DADA2. The features were classified at 99% identity using QIIME2. Taxonomy determination was carried out with SILVA and NT-16S. The alpha (α) diversity, including Shannon and Simpson index, was calculated with QIIME2. Also, the beta (β) diversity was acquired with QIIME2 and presented with Principal coordinate analysis (PCoA) based on Bray–Curtis dissimilarity and Principal Component Analysis (PCA). The functional prediction was based on the Kyoto Encyclopedia of Genes and Genomes (KEGG) and performed with PICRUSt (v1.1.2).

### Metagenomics

A total of 25 stool samples (10 H; 15 LUAD) were subjected to metagenomics. The DNA extraction and PCR amplification were as 16s-rRNA gene sequencing. The metagenome was fragmented, tagged, and quantified to generate the pooled library and paired-end reads. Then, low-quality paired-end reads were removed, and high-quality paired-end reads were mapped to the human genome to filter out chimeric sequences. The left high-quality paired-end reads were aligned using IGC bowtie2 (v 2.3.0) and mapped to metaphlan2 to perform species annotation. The α and β diversity, as well as functional prediction, were explored as 16s-rRNA gene sequencing.

### Plasma Metabolic Profiling

The methanol precipitation method was used for metabolite extraction. Firstly, 200 μL plasma was mixed with 400 μL methanol, vortexed for 60 s, sonicated for 10 mins, and incubated for 1 h to precipitate protein. Then, samples were centrifuged at 12000 g (10 mins, 4°C), and vacuum concentration drying. Next, 150 μL 2-chlorophenylalanine and 50 μL 80% methanol solution were added to the tube. All the operations are performed on ice.

The mixed liquids were filtrated to generate samples for the analysis of HPLC-MS. Detailed conditions for HPLC-MS analysis were reported in **Supplementary Material**. The peaks identification, peaks filtration, and peaks alignment were performed with XCMS (Version 3.7.1, https://xcmsonline.scripps.edu/) to obtain mass to charge ratio (m/z), retention time (rt), and intensity. Metabolite identification was performed with HMDB (Human Metabolome Database; http://www.hmdb.ca) and KEGG (Kyoto Encyclopedia of Genes and Genomes; https://www.kegg.jp) *via* matching mass, MS/MS information, and rt. The functional enrichment analysis of metabolites was based on MetaboAnalyst (Version:5.0; https://www.metaboanalyst.ca/).

### Statistical Analysis

Wilcoxon rank-sum test and Chi-square test were used for group comparison between two groups. The evenness and richness of the gut microbiome were assessed by the α diversity. The extent of the similarity of fecal microbial communities was assessed by the β diversity. With Galaxy online platform (http://huttenhower.sph.harvard.edu/galaxy/), Linear discriminant analysis (LDA) effect size (LEfSe) was applied to determine biomarker’s taxa. The cut-off value was the log value> 3.0 and Wilcoxon rank-sum test: P< 0.01. The biomarker’s taxon at the genus level was subjected to the logistic regression analysis to develop a diagnostic model. The diagnostic power of the model was estimated with the receiver operating characteristic curve (ROC) by comparing the area under the ROC (AUC) and the calibration plots. The correlation between the two indexes was calculated using the Spearman analysis. The canonical correspondence analysis (CCA) was performed to investigate the effect of clinical parameters on the distribution of the gut microbiome.

In metabonomics analysis, if a variable is missing in more than 80% of samples in each group, it should be eliminated; if the variable is missing in below 80% of all samples, the missing data should be replaced with the minimum value. The way for batch normalization was the total peak area normalization method; the standardization method was Par-scaling. After data processing, using the SIMCA-P (Version: 11.5), multivariate statistical analyses were executed, including the unsupervised analysis method: PCA, and supervised analysis methods: partial least-squares discrimination analysis (PLS-DA) and orthogonal partial least-squares discrimination analysis (OPLS-DA). In OPLS-DA, we calculated variable importance in projection (VIP). The differential metabolites were defined as FDR (False discovery rate) <0.05; Log2FC (Fold change) >1; VIP> 1. Functional enrichment analysis of metabolites was implemented with MetaboAnalyst 5.0 (https://www.metaboanalyst.ca/MetaboAnalyst/home.xhtml). Benjamini–Hochberg procedure was used to calculate the FDR value.

## Results

### 16s-rRNA Gene Sequencing

#### The Assessment of the α and β Diversity

**Figure 1:**
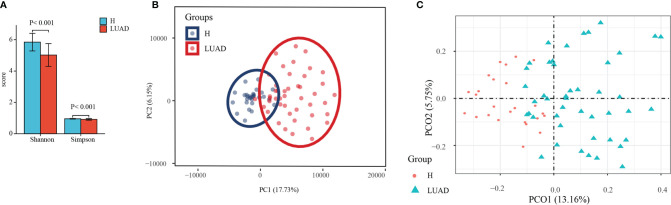
The assessment of the α and β diversity. **(A)** The α diversity was evaluated by Shannon and Simpson index. **(B)** The β diversity was evaluated by PCA analysis. **(C)** The β diversity was evaluated by PCoA analysis. H, healthy individuals; LUAD, Lung adenocarcinoma; PCA, Principal Component Analysis; PCoA, Principal coordinate analysis.

A total of 6,043,552 raw reads were acquired from 75 stool samples (30 H; 45 LUAD). In all, 9716 features were identified (7073 ± 112 features/sample). To characterize the diversity of the bacterial community, we assessed the α diversity for each sample. As shown in **Figure 1A**, the Shannon (P<0.001) and Simpson (P<0.001) indexes of the LUAD groups were significantly lower than that of the H group. In addition, we also took advantage of the Observed species, ACE, and Chao1 index to reflect the α diversity and got similar results (**Supplementary Figure 1A**). The curve of each sample in rarefaction analysis based on the Shannon index approached saturation, suggesting the sequencing depth was enough (**Supplementary Figure 1B**). To measure the extent of the similarity of fecal microbial communities, we presented the β diversity with PCA and PCoA analysis. In PCA and PCoA analysis, samples in different groups displayed significantly tighter clustering (**Figures 1B, C**).

#### Taxonomy Comparison of the Gut Microbiome

**Figure 2:**
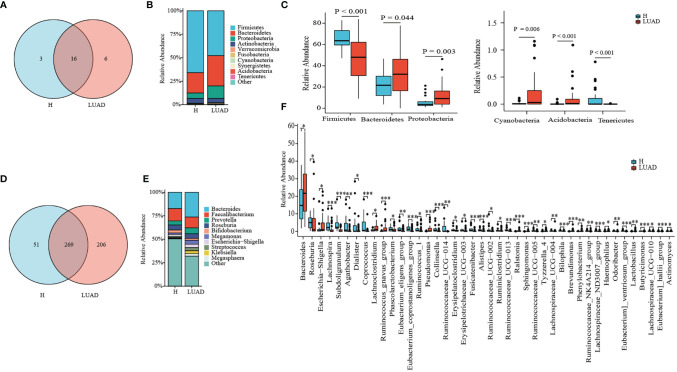
Taxonomy comparison of gut microbiome. **(A)** 19 and 22 phyla were identified in H group and LUAD group, respectively. **(B)** The distribution of top 10 phyla in two groups. **(C)** Differential phyla between two groups. **(D)** 320 and 475 genera were identified in H group and LUAD group, respectively. **(E)** The distribution of the top 10 genera in two groups. **(F)** Differential genera between two groups. *P < 0.05, **P < 0.01, ***P < 0.001.

Then, we analyzed the composition and structure of the gut microbiome at phylum, class, order, family, and genus levels. There were 19 and 22 phyla identified in the H and LUAD groups at the phylum level, respectively (**Figure 2A**). The top three abundant phyla in the LUAD group were Firmicutes (48.67%), Bacteroidetes (32.92), and Proteobacteria (12.61) (**Figure 2B**). Compared to the H group, the abundance of Bacteroidetes (P= 0.044), Proteobacteria (P= 0.003), Cyanobacteria (P= 0.006), and Acidobacteria (P< 0.001) were increased, whereas that of Firmicutes (P< 0.001), and Tenericutes (P< 0.001) were decreased (**Figure 2C** and **Supplementary Table 1**).

At genus levels, 475 genera were found in the LUAD group and 320 in the H group (**Figure 2D**). In the LUAD group, the most abundant genus was Bacteroides (25.06%), followed by Faecalibacterium (11.00%) and Prevotella (5.94%) (**Figure 2E**). There were significant differences in the proportion of 42 genera between the H and LUAD groups (**Figure 2F** and **Supplementary Table 1**).

#### The Influence of Clinical Parameters on the Distribution of Gut Microbiome

**Figure 3:**
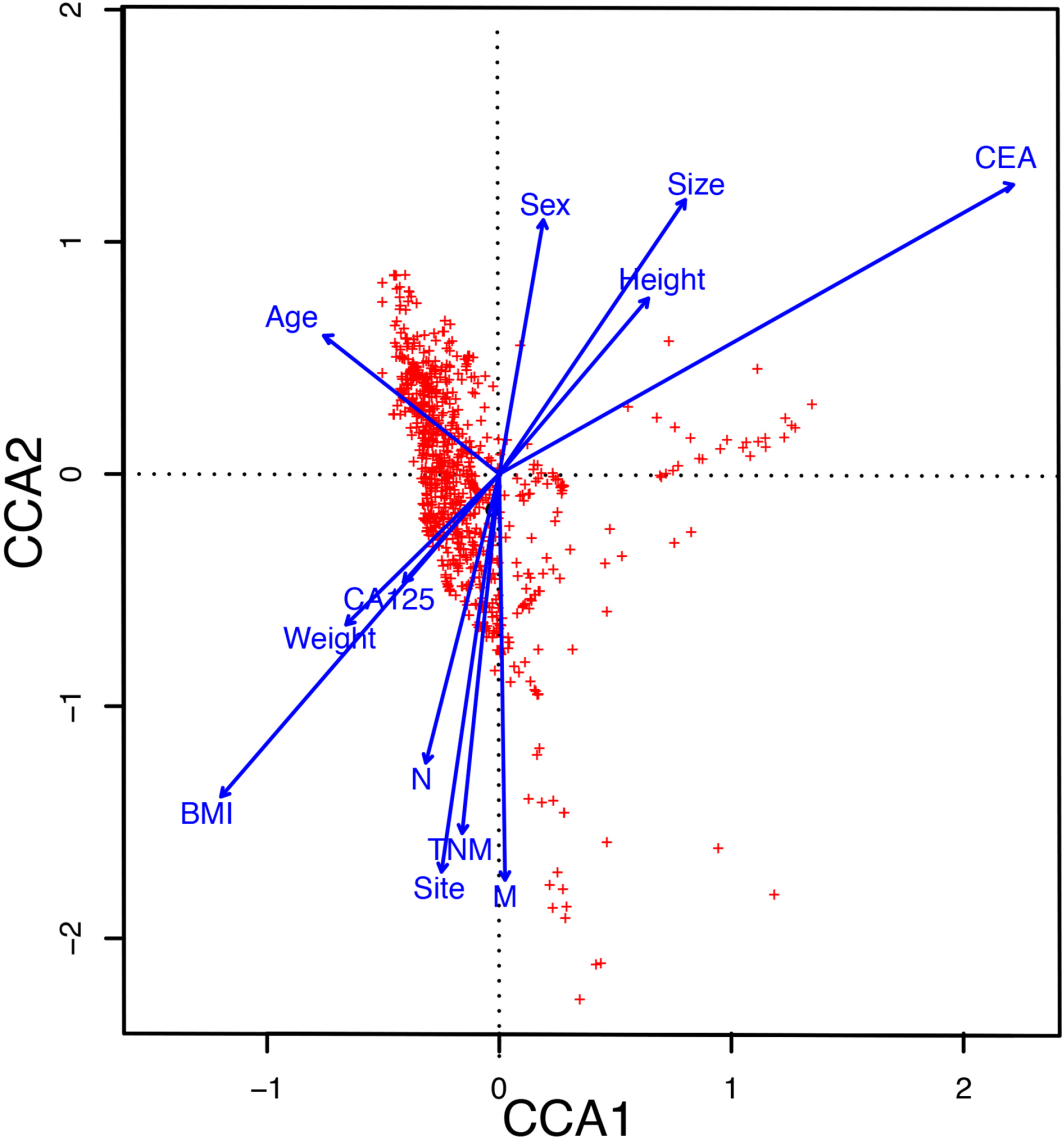
The CCA analysis revealed the influence of clinical parameters on the distribution and structure of the gut microbiome. CCA, The canonical correspondence analysis; CEA, Carcinoembryonic antigen; BMI, Body Mass Index.

To explore the influence of clinical parameters on the distribution and structure of the gut microbiome, we implemented CCA analysis, which could reflect the overall relationship between two groups of variables. The CCA analysis revealed that Carcinoembryonic antigen (CEA), Body Mass Index (BMI), and metastasis had a significant effect on the distribution of the gut microbiome (**Figure 3**).

#### Identification of Biomarker’s Taxa and Construction of a Diagnostic Model

**Figure 4:**
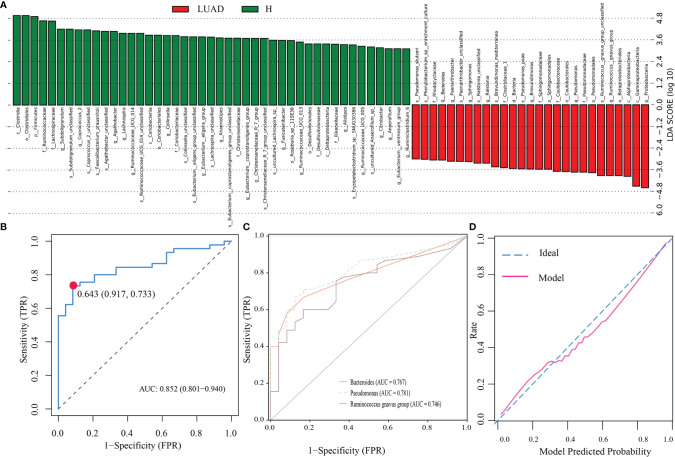
Identification of biomarker’s taxa and construction of a diagnostic model. **(A)** The LefSe analysis identified taxa meeting thresholds: P < 0.01 and LDA > 3. **(B)** The ROC of the diagnostic model. **(C)** The ROC of three biomarker’s taxa. **(D)** The calibration plot of the diagnostic model. LsfSe, Linear discriminant analysis (LDA) effect size; ROC, The receiver operating characteristic curve; AUC, the area under the ROC.

For investigating the biomarker’s taxa between those two groups, we performed LefSe analysis. As shown in **Figure 4A**, at genus levels, seven floras were markedly enriched in the LUAD group, including Bacteroides, Phenylobacterium, Sphingomonas, Ralstonia, Brevundimonas, Pseudomonas, and Ruminococcus gnavus group. Then, we applied the logistic regression analysis to further analyze those seven genera and screen out three genera as the biomarker’s taxa (Bacteroides, Pseudomonas, and Ruminococcus gnavus group) to construct a diagnostic model for LUAD. The diagnostic formula was presented as follows.


$$\text{Y}=\frac{1}{1+{\text{e}}^{-\left(-1.4431+0.0414*\text{Bacteroides}+4.7998*\text{Pseudomonas}+0.5941*\text{Ruminococcus gnavus group}\right)}}$$

To evaluate the accuracy of the model, we depicted the ROC and the calibration plots. The AUC of the model was 0.852 (**Figure 4B**), more excellent than that of Bacteroides (0.767), Pseudomonas (0.781), and Ruminococcus gnavus group (0.746) (**Figure 4C**). In addition, the calibration plot demonstrated that the model curve and ideal curve could fit well (**Figure 4D**).

### Metagenomics

**Figure 5:**
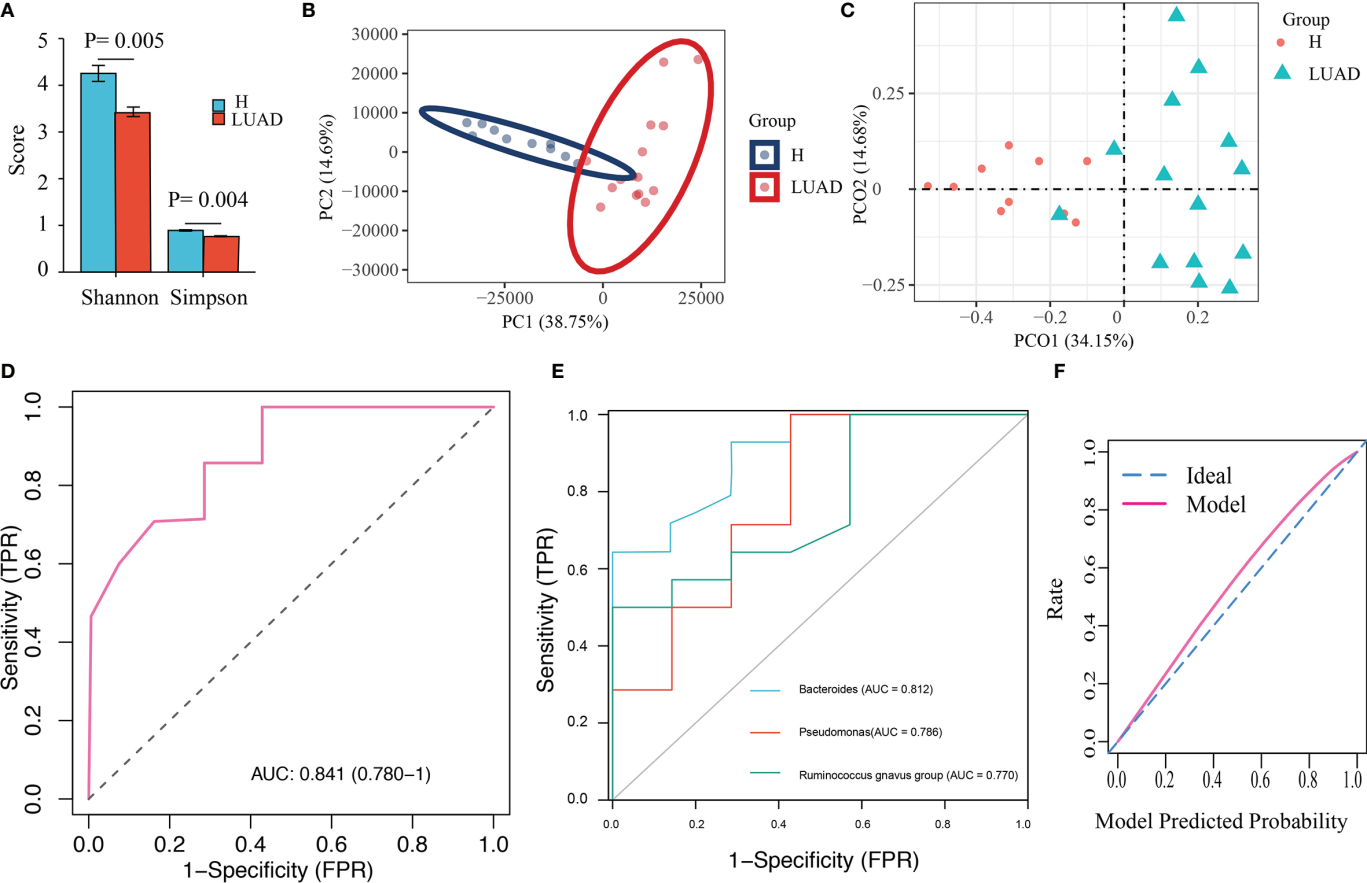
Validation of the diagnostic model with Metagenomics. **(A)** The α diversity is evaluated by Shannon and Simpson index. **(B)** The β diversity was evaluated by PCA analysis. **(C)** The β diversity was evaluated by PCoA analysis. **(D)** The ROC of the diagnostic model. **(E)** The ROC of three biomarker’s taxa. **(F)** The calibration plot of the diagnostic model.

Next, we analyzed 25 fecal samples (H: 10; LUAD: 15) with metagenomics to confirm the results of 16s-rRNA gene sequencing. In line with previous findings, the Shannon (P= 0.005) and Simpson (P= 0.004) indexes in the LUAD group were decreased (**Figure 5A**). Moreover, we also pictured the PCA and PCoA analysis. The results were presented in **Figures 5B**, **C**. At the phylum level, the top three most abundant bacteria in the LUAD group were Firmicutes, Bacteroidetes, and Proteobacteria (**Supplementary Figure 2A**). At genus levels were Bacteroides, Faecalibacterium, and Prevotella (**Supplementary Figure 2B**). We also analyzed the composition of the gut microbiome at a species level, and the results were presented in **Supplementary Figure 2D**.

We developed a diagnostic model with three indicator species (Bacteroides, Pseudomonas, and Ruminococcus gnavus group). Herein, we extracted the abundance of those three bacteria to validate the diagnostic model. Similar to previous results, the abundance of Bacteroides (P< 0.001), Pseudomonas (P= 0.037), and Ruminococcus gnavus group (P= 0.048) in the LUAD group were enhanced (**Supplementary Figure 2C**). As a result, the AUC in metagenomics was 0.841 (**Figure 5D**), higher than that of those three genera (Bacteroides: 0.812, Pseudomonas: 0.786, and Ruminococcus gnavus group: 0.770) (**Figure 5E**). The calibration plot was presented in **Figure 5F**.

### Functional Properties of the Gut Microbiome

We predicted the functional properties of the gut microbiome with PICRUSt (v1.1.2) in both 16s-rRNA gene sequencing and metagenomics. There were 99 and 84 pathways identified between H and LUAD groups in 16s-rRNA gene sequencing and metagenomics, respectively (Wilcoxon rank-sum test: FDR< 0.05, **Supplementary Figures S3A, B**). Among them, 34 pathways were overlapping (**Supplementary Figure 3C**). Furthermore, most of those pathways were metabolic-related pathways. Then, we sought the relation between 34 pathways and 3 indicator species with the Spearman analysis, which demonstrated several pathways were highly related to those three genera (**Supplementary Figure 3D**).

### Plasma Metabolic Profiling

**Figure 6:**
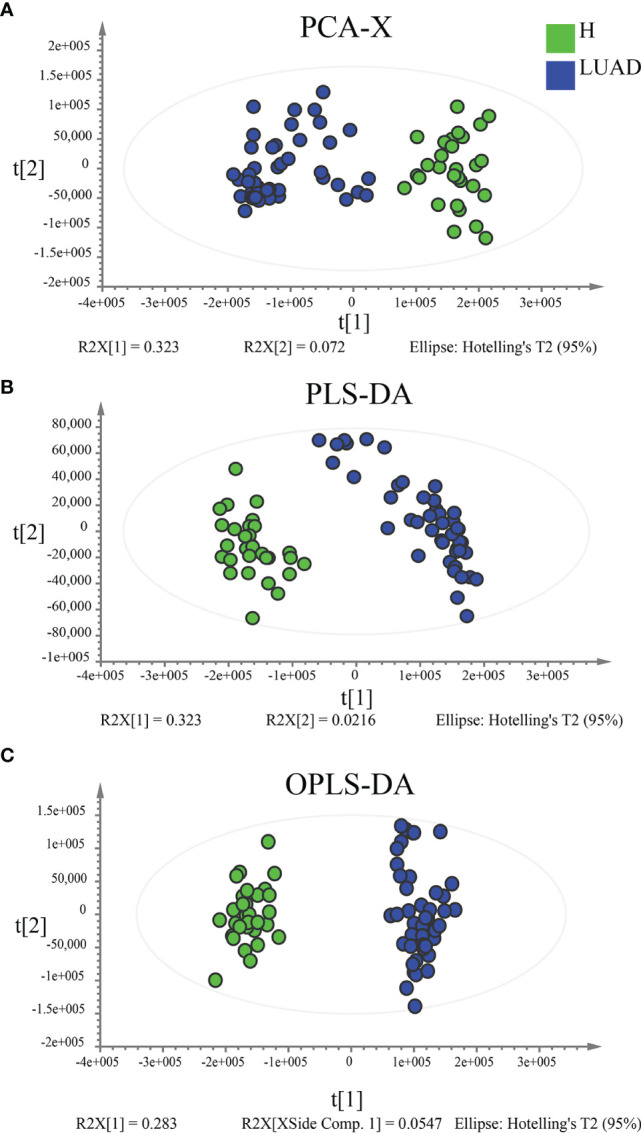
Multivariate statistical analyses to reveal the difference in plasma metabolic profiling between the H and LUAD groups. **(A)** Unsupervised analysis method: PCA. Supervised analysis methods: **(B)** PLS-DA and **(C)** OPLS-DA. PLS-DA, Partial least-squares discrimination analysis; OPLS-DA, Orthogonal partial least-squares discrimination analysis.

**Figure 7:**
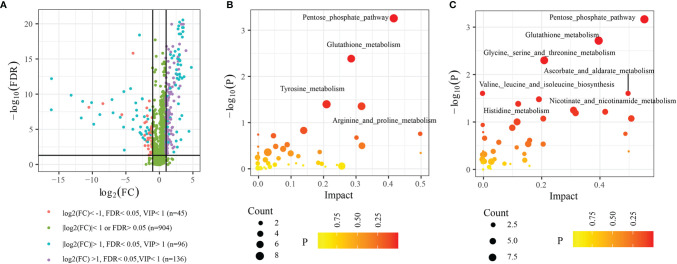
Differential metabolites and functional enrichment analysis. **(A)** 96 differential metabolites between H and LUAD group. **(B)** The metabolic pathways are enriched by 96 differential metabolites. **(C)** The metabolic pathways enriched by metabolites are highly related to the three biomarkers’ taxa. FDR, False discovery rate; VIP, Variable importance in projection; FC, Fold change.

In functional annotations of gut microbiota, we found numerous metabolic-related pathways were closely associated with gut microbiota, hinting that gut microbiota may function *via* regulating host metabolism in LUAD patients. Therefore, to prove the results, we investigated the plasma metabolic profiling of LUAD patients. A total of 1174 metabolites were determined, including 732 in positive ionization mode and 442 in negative ionization mode. Multivariate statistical analyses (PCA, PLS-DA, and OPLS-DA) were performed to obtain a global overview of the differences in metabolites between the two groups. As can be seen in **Figures 6A–C**, samples in different groups were well distinguished and clustered. Subsequently, we identified 96 differential metabolites with the cut-off value: FDR< 0.05; Log2FC> 1; VIP> 1 (**Figure 7A**), and 4 metabolic pathways enriched by them, including Pentose phosphate pathway (P< 0.001), Glutathione metabolism (P= 0.012), Tyrosine metabolism (P= 0.037), and Arginine and proline metabolism (P= 0.042) (**Figure 7B**).

### Correlation Analysis Between Plasma Metabolism and Biomarker’s Taxa

For further exploring the correlation between plasma metabolism and gut microbiome, we performed the Spearman analysis. The thresholds were set as |cor|> 0.40 and P< 0.05. The results showed that 102 plasma metabolites were highly related to three biomarkers’ bacteria (**Supplementary Table 2**), among which, 72 plasma metabolites were differential metabolites between the H and LUAD groups. Furthermore, functional enrichment analysis indicated that seven metabolic pathways were enriched by 102 plasma metabolites, such as the Pentose phosphate pathway (P< 0.001), Glutathione metabolism (P< 0.001), Glycine, serine, and threonine metabolism (P= 0.004), Valine, leucine, and isoleucine biosynthesis (P= 0.024), Ascorbate and aldarate metabolism (P= 0.024), Nicotinate and nicotinamide metabolism (P= 0.031), and Histidine metabolism (P= 0.039) (**Figure 7C**).

## Discussion

The study found that the profile of gut microbiota and the plasma metabolic profiling in LC patients were significantly different from those in healthy individuals. Moreover, the Spearman analysis showed that gut microbes were highly related to multiple plasma metabolisms, meaning that gut microbes may participate in regulating metabolism-related pathways in LUAD patients. Gut microbes are an essential part of the intestinal microenvironment (8). They interact with host cells in several ways. For example, they provided the pathogen-associated molecular patterns (PAMPs) linking to Toll-like receptors on the surface of the intestinal epithelial cells to activate innate-adaptive immunity (20, 21). Thereby, the immune cells secrete antimicrobial peptides, inflammatory factors, and immunoglobulins to regulate the immune response (20, 22). Moreover, regulating the production of metabolites is also a vital way that commensal bacteria function (23). For instance, gut microbes could modulate the production of short-chain fatty acids, which can enter the lung tissue through blood circulation and affect the differentiation and maturation of immune cells in lung tissue (23, 24).

Previously, a study reported that in LC patients, the composition and structure of the gut microbiome had a significant change. The diversity of intestinal flora in LC patients was significantly lower than that in healthy people (16, 24). At the same time, the relative abundance of beneficial bacteria such as Bifidobacterium and Lactobacillus decreased significantly, and the relative abundance of harmful bacteria such as Enterobacter and Streptococcus increased significantly (16, 24). In addition, the changes in relative abundance of the gut microbiome were closely related to tumor markers, such as CEA. Meanwhile, the gut microbiome could influence the therapeutic effect of LC. Patients undergoing immunotherapy with a high response rate of immunotherapy had high microbial diversity and a proportion of beneficial bacteria (18, 25). Furthermore, after being treated with the antibiotic, the homeostasis of intestinal flora was destroyed, and the diversity of intestinal flora decreased, leading to a low response rate (25). Although emerging evidence demonstrated the linking of the gut microbiome to LC and cancer therapy, their detailed role in LC has not been fully explicated.

In the study, we investigated the gut microbiota profile in LC patients with 16s-rRNA gene sequencing and metagenomics. Consistent with the previous findings, we found the Shannon and Simpson index in LUAD groups was significantly lower than that of the H group, indicating that the richness and evenness of the bacterial community in LUAD patients were lower than in healthy individuals. Moreover, the PCA and PCoA analysis demonstrated that the similarity of fecal microbial communities of LUAD patients was significantly different from that of healthy individuals. In LUAD patients, the top three abundant phyla were Firmicutes, Bacteroidetes, and Proteobacteria. The top three abundant genera were Bacteroides, Faecalibacterium, and Prevotella, which were in line with the study reported by Zhang et al. (16) We identified three genera (Bacteroides, Pseudomonas, and Ruminococcus gnavus group) as the biomarkers’ taxa with logistic regression analysis and constructed a diagnostic model. The AUCs of the diagnostic model were > 0.80 in both 16s-rRNA gene sequencing and metagenomics. Additionally, the calibration plots demonstrated consistency between the prediction by the model and the actual observation. All data suggested that the established diagnostic model is suitable for the diagnosis of LUAD. Bacteroides are the most common bacteria in healthy individuals, and the abundance of Bacteroides in LC patients is enhanced (16), which was confirmed by our findings. In the human body, Bacteroides are like a double-edged sword. On the one hand, they can protect the intestinal mucosal barrier, maintain intestinal homeostasis, mediate carbohydrate metabolism and induce T lymphocyte-dependent immune response (26, 27). On the other hand, it can also lead to immune escape, the production of endotoxin, and local inflammation in the intestine. Accumulating evidence has illustrated that Bacteroides were closely associated with the tumorigenesis and development of cancers (28–30). For example, Bacteroides could promote colon cancer progression *via* inducing the stemness of colon cancer cells and activating RHEB/mTOR signaling pathway (29). In addition, Bacteroides could also increase the expression of cyclooxygenase 2 and the release of prostacyclin 2 to induce local inflammation of intestinal mucosa and regulate the survival and proliferation of tumor cells (30). Up to now, no research has reported the specific effect of Bacteroides on lung cancer, so a lot of research is still needed to explore the role and mechanism of Bacteroides on lung cancer. Pseudomonas is a Gram-negative, opportunistic, bacterial pathogen associated with a wide range of infections. Cancer patients are more vulnerable to invasive infection, due to ulcerative lesions in mucosal surfaces and immune suppression (31). The infection of Pseudomonas could increase E-cadherin expression in colon cancer to promote cancer development (32). In the study, we found that the abundance of Pseudomonas in LUAD patients was ascending, especially in patients at TNM stage III/IV (**Supplementary Figure 4**). However, many studies reported that Pseudomonas could secret multiple substances (Cyclodipeptides, Phenazine-1-carboxamide, Fucoxanthinol, etc.) to anti-cancer by inducing the apoptosis of cancers (33–35). Thence, it remains a depth exploration. Ruminococcus gnavus is an anaerobic Gram-positive bacterial pathogen that can be found in the gastrointestinal tract of animals and humans (36). It could stimulate intestinal mucosal inflammation, activate local immunity, and regulate bile acid metabolism (36–38). Plus, orally administered Ruminococcus gnavus may enhance regulatory T-cell counts and short-chain fatty acids production (39). However, up to now, there has been no research reporting the role of Ruminococcus gnavus on LC.

We identified 34 overlapping differential pathways in 16s-rRNA gene sequencing and metagenomics. Those 34 pathways were the main metabolic-related pathways and highly related to the three biomarkers’ taxa, hinting that those three biomarkers’ taxa may participate in the regulation of host metabolism. For testing this conjecture, we analyzed the plasma metabolic profiling of LUAD patients. Between the H and LUAD groups, 96 differential metabolites were determined, mainly involved in four metabolic pathways. Then, we performed the Spearman analysis and found 102 plasma metabolites were closely associated with the three biomarkers’ taxa. Moreover, those 102 plasma metabolites significantly enriched seven metabolic pathways. Interestingly, two metabolic pathways (Pentose phosphate pathway, Glutathione metabolism) were significantly enriched in each part of the study, suggesting that gut microbes, especially those three biomarkers’ taxa, play a vital role in pentose phosphate pathway and glutathione metabolism. Pentose phosphate pathway, beginning with glucose 6-phosphate, is a way of oxidative decomposition of glucose and a branch from glycolysis (40). Unlike glycolysis, the Pentose phosphate pathway does not supply energy. Instead, it mainly provides NADPH and ribose 5-phosphate (R5P), which is critical for managing DNA damage response, metabolism, proliferation, and metastasis of cancer cells (40, 41). In LC, studies showed that regulating the Pentose phosphate pathway could contribute to LC cells’ growth and invasion (42, 43). The Spearman analysis revealed that nine metabolites in the Pentose phosphate pathway were highly related to three biomarker’s taxa, including alpha-D-Glucose 6-phosphate, 2-Deoxy-D-ribose 1-phosphate, 2-Deoxy-D-ribose 5-phosphate, D-Ribose, D-Glyceraldehyde 3-phosphate, D-Ribulose 5-phosphate, D-Glycerate, beta-D-Glucose 6-phosphate, and 6-Phospho-D-gluconate. In addition, nine metabolites in Glutathione metabolism were also highly associated with three biomarker’s taxa, such as glutathione, glutamate, and cysteine. Previous research has proved that Bacteroides could influence the homeostasis of host health and the production of glutathione (44, 45). Glutathione is a ubiquitous anti-oxidant involved in anti-oxidation, exogenous detoxifying substances, maintaining cysteine levels, maturation of protein iron-sulfur clusters, and regulation of redox signal-related transcription factors (46, 47). Glutathione plays a dual role in tumor cells: at normal levels, glutathione could clear carcinogens maintaining normal cell survival; excessive glutathione protects and promotes cancer cell proliferation, metastasis, and resistance to chemotherapeutic drugs (46–49). Animal experiments revealed that intestinal flora could regulate glutathione metabolism in mice, and this regulation can promote the production of oxidants and promote intestinal endothelial cell abscission and local inflammation (50). In LC, glutathione could affect the growth, metastasis, survival, and drug sensitivity of cancer cells. Glutamate was a product of the metabolism of glutathione (51–54). A study showed that the level of glutamate in lung cancer was down-regulated (55). Furthermore, glutamate suppressed tumor growth and prolonged survival of mice with LC (56).

Limitations: 1. We only used 45 LUAD patients to develop a diagnostic model and verified it with 15 LUAD patients. Therefore, a large-scale clinical trial was needed. 2. All data were obtained from costly and time-consuming omics technology, leading to hardly any popularization in clinical applications. Hence, a simple and efficient detection method is still needed. 3. In the study, we excluded individuals with other acute and chronic diseases influencing the composition of the gut microbiome, which may lead to selection bias. 4. No basic experiments were conducted to investigate the specific role of gut microbiota, and further investigation is demanded. 5. Due to only using healthy individuals as the control group and lack of other types of cancer, Whether the three biomarkers’ taxa were specific for LUAD patients still requires further investigation.

## Conclusion

In conclusion, in this study, we investigated the composition and structure of the gut microbiome in LUAD patients with 16s-rRNA gene sequencing and screened out the taxa of three biomarkers to construct a diagnostic model, which was confirmed with metagenomics. The ROC demonstrated the model was accurate. Then, we predicted the potential functions of the gut microbiome in LUAD patients and verified the results with plasma metabonomics. Gut microbes may participate in regulating metabolism-related pathways in LUAD patients, such as the pentose phosphate pathway and glutathione metabolism.

## Data Availability Statement

The datasets presented in this study can be found in online repositories. The names of the repository/repositories and accession number(s) can be found below: MetaboLights, http://www.ebi.ac.uk/metabolights: MTBLS3865; and EBI metagenomics, http://www.ebi.ac.uk/metagenomics: PRJEB48980.

## Ethics Statement

The studies involving human participants were reviewed and approved by The ethics committee of Guangfu hospital. The patients/participants provided their written informed consent to participate in this study.

## Author Contributions

XW and HX designed the research. SW, HC, and HY conducted the experiments. SW and FB analyzed the data. SW, HC, and FB wrote the manuscript. All authors contributed to the article and approved the submitted version.

## Conflict of Interest

The authors declare that the research was conducted in the absence of any commercial or financial relationships that could be construed as a potential conflict of interest.

## Publisher’s Note

All claims expressed in this article are solely those of the authors and do not necessarily represent those of their affiliated organizations, or those of the publisher, the editors and the reviewers. Any product that may be evaluated in this article, or claim that may be made by its manufacturer, is not guaranteed or endorsed by the publisher.

## Supplementary Material

Supplementary Figure 1 **(A)** The α diversity is evaluated by observed species, ACE, and Chao1. **(B)** Rarefaction analyses based on the Shannon index. H, healthy individuals; LUAD, Lung adenocarcinoma. ***P < 0.001.

Supplementary Figure 2 Taxonomy comparison of the gut microbiome with Metagenomics. **(A)** The distribution of the top 10 phyla in two groups. **(B)** The distribution of the top 10 genera in two groups. **(C)** The differential distribution of three biomarkers’ taxa between two groups. **(D)** The distribution of the top 10 species in two groups.

Supplementary Figure 3 Functional properties of the gut microbiome. **(A)** 99 pathways were identified between H and LUAD groups in 16s-rRNA gene sequencing. **(B)** 84 pathways were identified between H and LUAD groups in metagenomics. **(C)** The distribution of 34 overlapping pathways in all samples. **(D)** The relation of three biomarker’s taxa to 34 overlapping pathways. *P < 0.05, **P < 0.01.

Supplementary Figure 4 The differential distribution of three biomarkers’ taxa **(A)** between Age ≥ 60 and < 60, **(B)** between female and male, **(C)** between BMI ≥ 24 and < 24, **(D)** between TNM stage I/II and TNM stage III/IV. BMI, Body Mass Index. *P < 0.05, **P < 0.01.
