# Supplementary figures and images for: Gut Microbiome Was Highly Related to the Regulation of Metabolism in Lung Adenocarcinoma Patients
Source: Front Oncol. 2022 May 3;12:790467. doi: 10.3389/fonc.2022.790467 (PMC9113755; doi:10.3389/fonc.2022.790467)

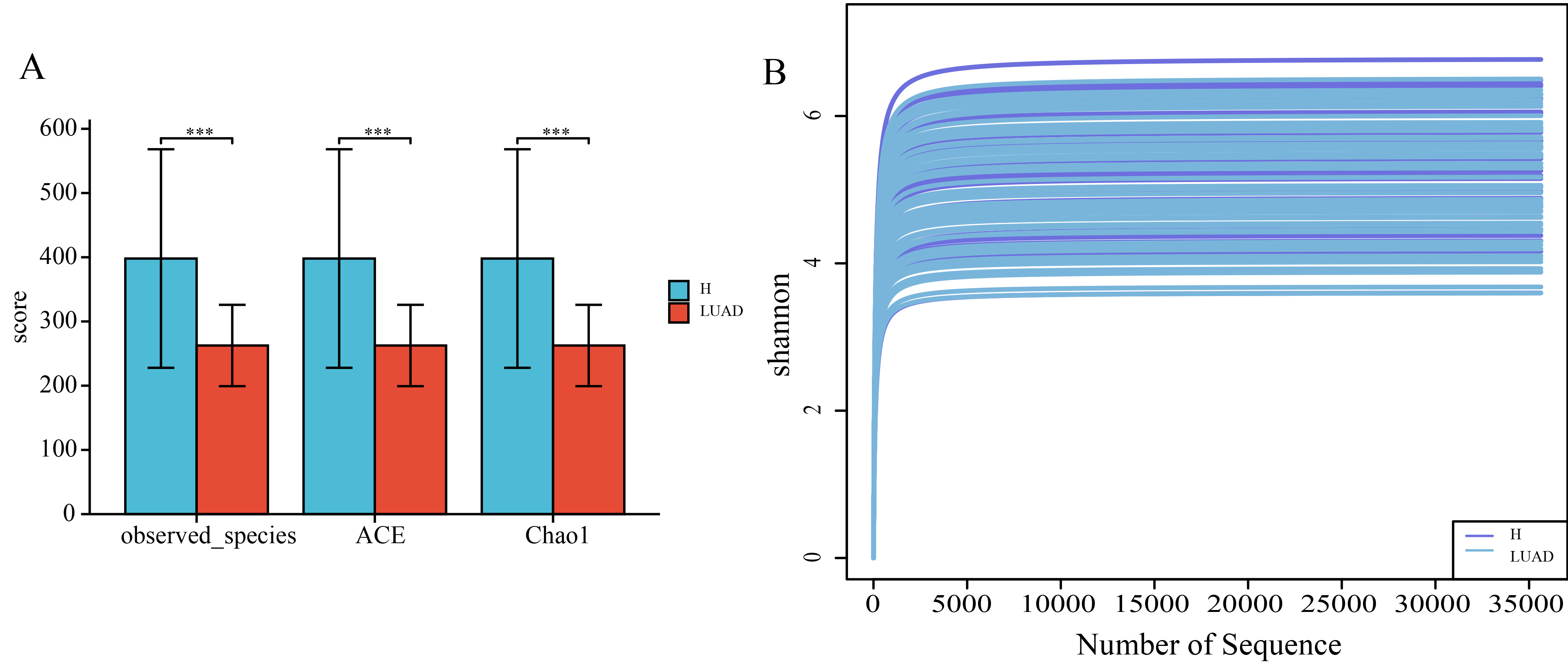

Supplement: Supplementary Figure 1 — (A) The α diversity is evaluated by observed species, ACE, and Chao1. (B) Rarefaction analyses based on the Shannon index. H, healthy individuals; LUAD, Lung adenocarcinoma. ***P < 0.001. [file Image_1.jpeg]

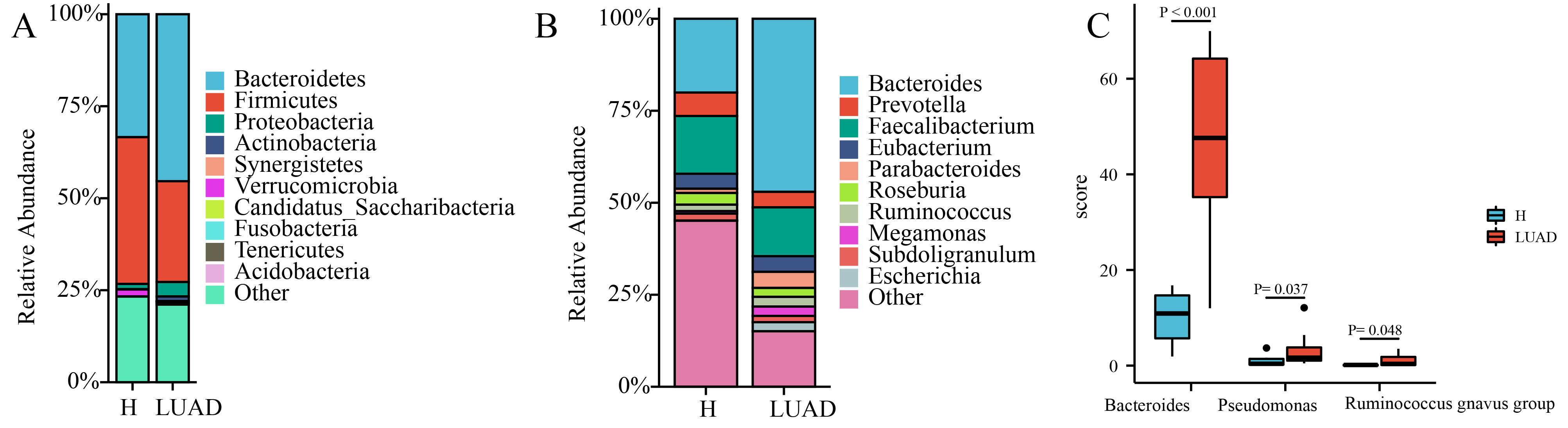

Supplement: Supplementary Figure 2 — Taxonomy comparison of the gut microbiome with Metagenomics. (A) The distribution of the top 10 phyla in two groups. (B) The distribution of the top 10 genera in two groups. (C) The differential distribution of three biomarkers’ taxa between two groups. (D) The distribution of the top 10 species in two groups. [file Image_2.jpeg]

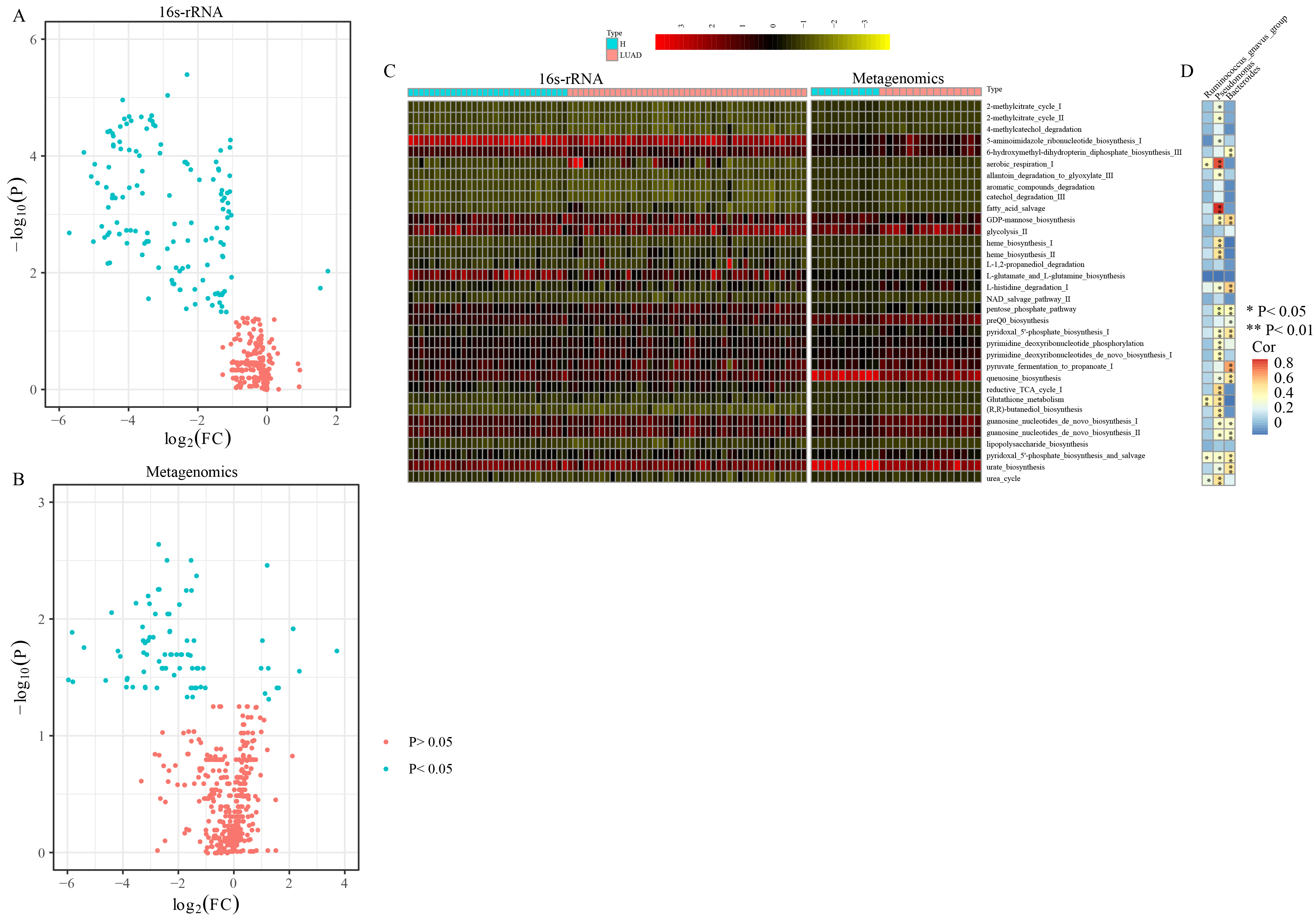

Supplement: Supplementary Figure 3 — Functional properties of the gut microbiome. (A) 99 pathways were identified between H and LUAD groups in 16s-rRNA gene sequencing. (B) 84 pathways were identified between H and LUAD groups in metagenomics. (C) The distribution of 34 overlapping pathways in all samples. (D) The relation of three biomarker’s taxa to 34 overlapping pathways. *P < 0.05, **P < 0.01. [file Image_3.jpeg]

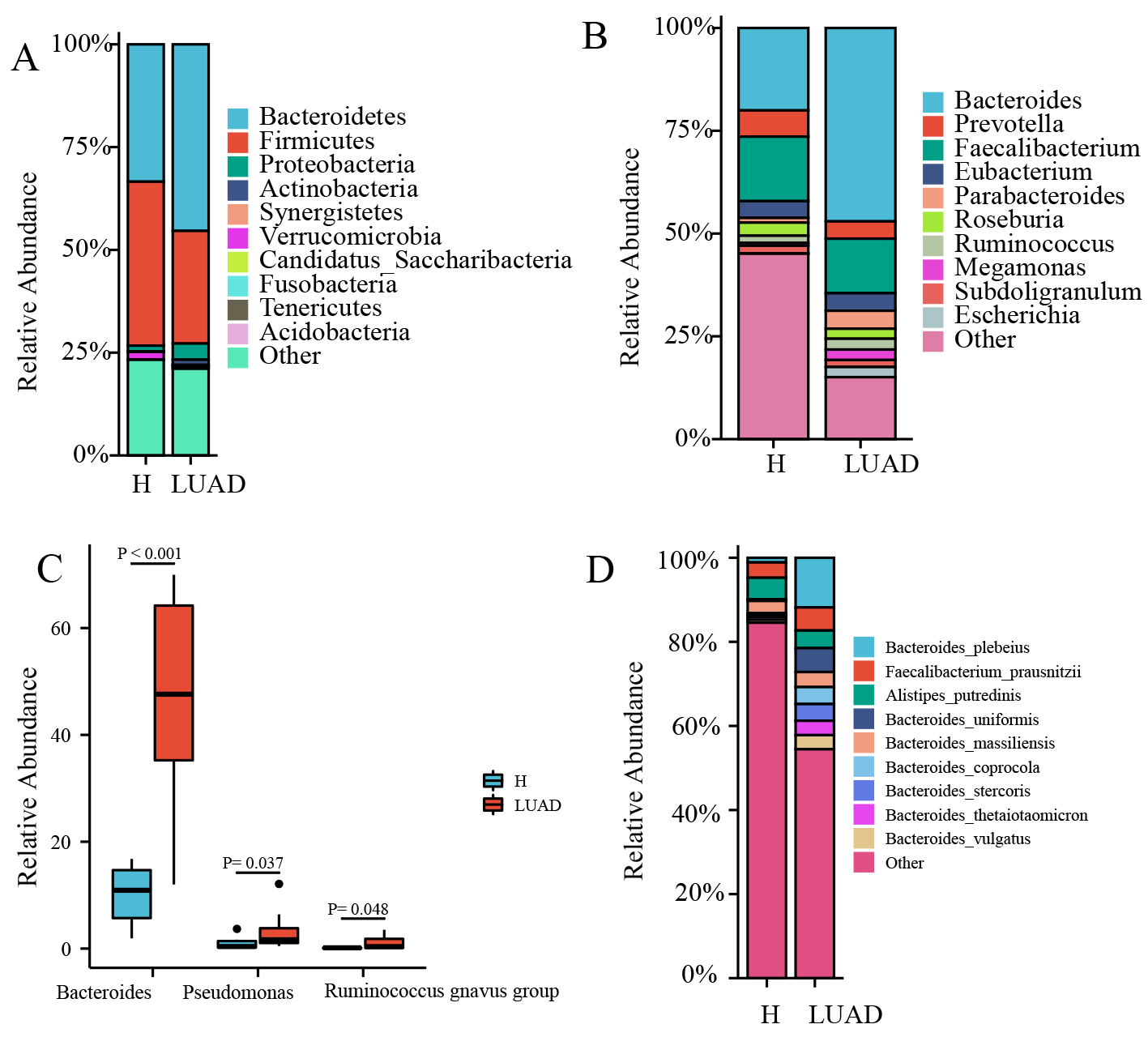

Supplement: Supplementary Figure 4 — The differential distribution of three biomarkers’ taxa (A) between Age ≥ 60 and < 60, (B) between female and male, (C) between BMI ≥ 24 and < 24, (D) between TNM stage I/II and TNM stage III/IV. BMI, Body Mass Index. *P < 0.05, **P < 0.01. [file Image_4.jpeg]
