# Supplementary material for: Gut Microbiome Was Highly Related to the Regulation of Metabolism in Lung Adenocarcinoma Patients
Source: Front Oncol. 2022 May 3;12:790467. doi: 10.3389/fonc.2022.790467 (PMC9113755; doi:10.3389/fonc.2022.790467)
Supplement: Supplementary file 5 [file Table_1.docx]

Supplementary Table 1. Taxonomy comparison of gut microbiome at phylum and genus level.

| Phylum | H | LUAD | P |
| --- | --- | --- | --- |
| Firmicutes | 64.613±10.356 | 47.118±18.768 | *** |
| Bacteroidetes | 23.165±12.136 | 32.484±19.985 | * |
| Proteobacteria | 5.677±5.304 | 13.617±16.003 | ** |
| Cyanobacteria | 0.015±0.026 | 0.188±0.307 | ** |
| Acidobacteria | 0.006±0.018 | 0.122±0.378 | *** |
| Tenericutes | 0.108±0.196 | 0.001±0.002 | *** |
| Genus | H | LUAD | P |
| Bacteroides | 16.515±10.965 | 23.947±14.253 | * |
| Roseburia | 5.150±3.553 | 4.687±8.091 | * |
| Escherichia-Shigella | 2.174±4.649 | 3.433±5.253 | * |
| Lachnospira | 3.235±3.256 | 1.164±2.523 | *** |
| Subdoligranulum | 3.972±3.884 | 0.724±1.070 | *** |
| Agathobacter | 3.234±4.831 | 0.754±2.242 | ** |
| Dialister | 3.469±7.116 | 0.565±1.497 | * |
| Coprococcus | 3.288±4.861 | 0.395±1.453 | *** |
| Lachnoclostridium | 0.650±0.647 | 1.775±1.906 | * |
| Ruminococcus]_gnavus_group | 0.240±0.666 | 1.744±3.140 | *** |
| Phascolarctobacterium | 1.379±1.348 | 0.898±1.269 | * |
| Eubacterium]_eligens_group | 1.600±2.109 | 0.415±0.782 | ** |
| Eubacterium]_coprostanoligenes_group | 1.494±1.349 | 0.440±0.997 | *** |
| Ruminococcus_1 | 1.087±1.354 | 0.657±1.571 | * |
| Pseudomonas | 0.055±0.104 | 1.130±1.792 | *** |
| Collinsella | 1.697±2.831 | 0.215±0.600 | *** |
| Ruminococcaceae_UCG-014 | 1.762±3.175 | 0.009±0.018 | ** |
| Erysipelatoclostridium | 0.842±1.038 | 0.464±0.571 | * |
| Erysipelotrichaceae_UCG-003 | 0.684±1.130 | 0.487±1.077 | * |
| Fusicatenibacter | 1.058±1.337 | 0.242±0.514 | *** |
| Alistipes | 0.770±0.742 | 0.384±0.899 | *** |
| Ruminococcaceae_UCG-002 | 1.097±2.137 | 0.186±0.419 | * |
| Ruminiclostridium | 0.520±0.801 | 0.355±0.646 | * |
| Ruminococcaceae_UCG-013 | 0.782±0.806 | 0.166±0.243 | *** |
| Ralstonia | 0.017±0.029 | 0.390±0.880 | *** |
| Sphingomonas | 0.003±0.010 | 0.098±0.196 | *** |
| Ruminococcaceae_UCG-005 | 0.414±0.608 | 0.098±0.227 | ** |
| Tyzzerella_4 | 0.070±0.186 | 0.277±0.482 | * |
| Lachnospiraceae_UCG-004 | 0.181±0.216 | 0.212±0.833 | ** |
| Bilophila | 0.283±0.204 | 0.148±0.216 | *** |
| Brevundimonas | 0.008±0.016 | 0.283±0.645 | *** |
| Phenylobacterium | 0.017±0.043 | 0.255±0.537 | ** |
| Ruminococcaceae_NK4A214_group | 0.243±0.367 | 0.092±0.289 | ** |
| Lachnospiraceae_ND3007_group | 0.216±0.307 | 0.084±0.172 | *** |
| Haemophilus | 0.064±0.190 | 0.161±0.325 | * |
| Odoribacter | 0.109±0.141 | 0.134±0.591 | ** |
| Eubacterium]_ventriosum_group | 0.282±0.331 | 0.040±0.080 | *** |
| Lactobacillus | 0.025±0.050 | 0.161±0.394 | ** |
| Butyricimonas | 0.164±0.167 | 0.059±0.127 | *** |
| Lachnospiraceae_UCG-010 | 0.107±0.143 | 0.087±0.181 | * |
| Eubacterium]_hallii_group | 0.095±0.162 | 0.041±0.106 | ** |
| Actinomyces | 0.027±0.081 | 0.060±0.123 | * |

Notes: H: healthy individual; LUAD: Lung adenocarcinoma; Wilcoxon rank-sum test: * P< 0.05; ** P< 0.01, *** P< 0.001.
