# Supplementary material for: Gut Microbiome Was Highly Related to the Regulation of Metabolism in Lung Adenocarcinoma Patients
Source: Front Oncol. 2022 May 3;12:790467. doi: 10.3389/fonc.2022.790467 (PMC9113755; doi:10.3389/fonc.2022.790467)
Supplement: Supplementary file 6 [file Table_2.docx]

Supplementary Table 1. The metabolites highly related to three biomarker’s taxa.

| Genus | Metabolite | Cor | *P* |
| --- | --- | --- | --- |
| Bacteroides | C01073 | 0.630 | 0.003 |
| Bacteroides | C01094 | -0.595 | 0.007 |
| Bacteroides | C03299 | 0.593 | 0.007 |
| Bacteroides | C05577 | -0.587 | 0.008 |
| Bacteroides | C00504 | 0.580 | 0.009 |
| Bacteroides | C04874 | 0.576 | 0.009 |
| Bacteroides | C02216 | -0.576 | 0.009 |
| Bacteroides | C04556 | 0.561 | 0.012 |
| Bacteroides | C02537 | -0.556 | 0.013 |
| Bacteroides | C01993 | 0.543 | 0.016 |
| Bacteroides | C00847 | -0.527 | 0.020 |
| Bacteroides | C00051 | -0.522 | 0.021 |
| Bacteroides | C06425 | 0.515 | 0.023 |
| Bacteroides | C00311 | 0.510 | 0.025 |
| Bacteroides | C00114 | 0.509 | 0.025 |
| Bacteroides | C01151 | 0.504 | 0.027 |
| Bacteroides | C03150 | 0.500 | 0.029 |
| Bacteroides | C01507 | -0.493 | 0.031 |
| Bacteroides | C00121 | 0.492 | 0.032 |
| Bacteroides | C01672 | -0.490 | 0.033 |
| Bacteroides | C00036 | -0.486 | 0.034 |
| Bacteroides | C00019 | 0.483 | 0.036 |
| Bacteroides | C01880 | 0.479 | 0.037 |
| Bacteroides | C00355 | -0.475 | 0.039 |
| Bacteroides | C00363 | 0.473 | 0.040 |
| Bacteroides | C00672 | -0.471 | 0.041 |
| Bacteroides | C05281 | -0.469 | 0.042 |
| Bacteroides | C01752 | -0.469 | 0.042 |
| Bacteroides | C05574 | -0.468 | 0.043 |
| Bacteroides | C00785 | 0.464 | 0.0449 |
| Bacteroides | C00331 | 0.464 | 0.045 |
| Bacteroides | C00051 | 0.463 | 0.045 |
| Bacteroides | C00668 | -0.463 | 0.045 |
| Bacteroides | C00379 | 0.458 | 0.048 |
| Bacteroides | C05726 | 0.457 | 0.048 |
| Pseudomonas | C00354 | 0.948 | <0.001 |
| Pseudomonas | C01606 | 0.813 | <0.001 |
| Pseudomonas | C03044 | 0.811 | <0.001 |
| Pseudomonas | C00262 | 0.723 | <0.001 |
| Pseudomonas | C00360 | 0.720 | <0.001 |
| Pseudomonas | C16562 | 0.703 | <0.001 |
| Pseudomonas | C00345 | 0.694 | <0.001 |
| Pseudomonas | C02154 | 0.685 | 0.001 |
| Pseudomonas | C00118 | 0.664 | 0.001 |
| Pseudomonas | C02952 | 0.656 | 0.002 |
| Pseudomonas | C00049 | 0.632 | 0.003 |
| Pseudomonas | C06428 | 0.614 | 0.005 |
| Pseudomonas | C00025 | 0.609 | 0.005 |
| Pseudomonas | C00750 | 0.588 | 0.008 |
| Pseudomonas | C00109 | 0.565 | 0.011 |
| Pseudomonas | C04675 | 0.553 | 0.013 |
| Pseudomonas | C00074 | 0.546 | 0.015 |
| Pseudomonas | C00108 | 0.514 | 0.024 |
| Pseudomonas | C00548 | 0.491 | 0.032 |
| Pseudomonas | C00022 | 0.487 | 0.034 |
| Pseudomonas | C01934 | 0.482 | 0.036 |
| Pseudomonas | C00258 | 0.469 | 0.042 |
| Pseudomonas | C05711 | 0.460 | 0.047 |
| Ruminococcus_gnavus_group | C00725 | 0.774 | <0.001 |
| Ruminococcus_gnavus_group | C02147 | 0.693 | <0.001 |
| Ruminococcus_gnavus_group | C00673 | 0.692 | 0.001 |
| Ruminococcus_gnavus_group | C01026 | 0.674 | 0.001 |
| Ruminococcus_gnavus_group | C05984 | 0.674 | 0.001 |
| Ruminococcus_gnavus_group | C00248 | 0.668 | 0.001 |
| Ruminococcus_gnavus_group | C01120 | 0.668 | 0.001 |
| Ruminococcus_gnavus_group | C00311 | 0.667 | 0.001 |
| Ruminococcus_gnavus_group | C06949 | 0.663 | 0.001 |
| Ruminococcus_gnavus_group | C03415 | 0.651 | 0.002 |
| Ruminococcus_gnavus_group | C07083 | 0.645 | 0.002 |
| Ruminococcus_gnavus_group | C00073 | 0.644 | 0.002 |
| Ruminococcus_gnavus_group | C01993 | 0.639 | 0.003 |
| Ruminococcus_gnavus_group | C01094 | 0.631 | 0.003 |
| Ruminococcus_gnavus_group | C02090 | 0.626 | 0.004 |
| Ruminococcus_gnavus_group | C00052 | 0.621 | 0.004 |
| Ruminococcus_gnavus_group | C01081 | 0.616 | 0.004 |
| Ruminococcus_gnavus_group | C00818 | 0.615 | 0.005 |
| Ruminococcus_gnavus_group | C02703 | 0.608 | 0.005 |
| Ruminococcus_gnavus_group | C05730 | 0.591 | 0.007 |
| Ruminococcus_gnavus_group | C00191 | 0.591 | 0.007 |
| Ruminococcus_gnavus_group | C00043 | 0.587 | 0.008 |
| Ruminococcus_gnavus_group | C04871 | 0.583 | 0.008 |
| Ruminococcus_gnavus_group | C01752 | -0.580 | 0.009 |
| Ruminococcus_gnavus_group | C06044 | 0.569 | 0.010 |
| Ruminococcus_gnavus_group | C00315 | -0.557 | 0.013 |
| Ruminococcus_gnavus_group | C00233 | 0.542 | 0.016 |
| Ruminococcus_gnavus_group | C01829 | 0.541 | 0.016 |
| Ruminococcus_gnavus_group | C00250 | 0.538 | 0.017 |
| Ruminococcus_gnavus_group | C00199 | -0.529 | 0.019 |
| Ruminococcus_gnavus_group | C00092 | 0.526 | 0.020 |
| Ruminococcus_gnavus_group | C02083 | 0.524 | 0.021 |
| Ruminococcus_gnavus_group | C01879 | 0.521 | 0.022 |
| Ruminococcus_gnavus_group | C00097 | 0.513 | 0.024 |
| Ruminococcus_gnavus_group | C05143 | 0.512 | 0.024 |
| Ruminococcus_gnavus_group | C00383 | -0.510 | 0.025 |
| Ruminococcus_gnavus_group | C03519 | 0.504 | 0.027 |
| Ruminococcus_gnavus_group | C00147 | 0.504 | 0.027 |
| Ruminococcus_gnavus_group | C05608 | 0.501 | 0.028 |
| Ruminococcus_gnavus_group | C00864 | -0.501 | 0.028 |
| Ruminococcus_gnavus_group | C00095 | 0.500 | 0.029 |
| Ruminococcus_gnavus_group | C02480 | 0.497 | 0.030 |
| Ruminococcus_gnavus_group | C05145 | 0.495 | 0.031 |
| Ruminococcus_gnavus_group | C00671 | -0.484 | 0.035 |
| Ruminococcus_gnavus_group | C04874 | 0.481 | 0.037 |
| Ruminococcus_gnavus_group | C00025 | 0.479 | 0.037 |
| Ruminococcus_gnavus_group | C02915 | 0.478 | 0.038 |
| Ruminococcus_gnavus_group | C05601 | 0.472 | 0.041 |
| Ruminococcus_gnavus_group | C00315 | 0.471 | 0.041 |
| Ruminococcus_gnavus_group | C00153 | 0.470 | 0.042 |
| Ruminococcus_gnavus_group | C02670 | 0.462 | 0.046 |
| Ruminococcus_gnavus_group | C06867 | 0.656 | 0.002 |
